# Virtual sailing exercise enhances motor performance while maintaining stable mental and neurochemical profiles in healthy adults: a pilot study

**DOI:** 10.3389/fphys.2026.1789933

**Published:** 2026-05-07

**Authors:** Radietya Alvarabie, Elissa Burjawi, Gurdeep Sarai, Yang Yun, Oren Tirosh, Denny Meyer, Junhua Xiao

**Affiliations:** 1 Department of Biomedical, Health and Exercise Sciences, School of Health Sciences, Swinburne University of Technology, VIC, Hawthorn, Australia; 2 Northern Area Mental Health Service, Northern Health, Epping, VIC, Australia; 3 School of Health and Biomedical Sciences, Royal Melbourne Institute of Technology, Bundoora, VIC, Australia

**Keywords:** exercise, mental health, motor function, MRS, neurometabolite, sailing, virtual reality

## Abstract

**Objectives:**

While virtual exercise is emerging as a new approach for clinical application, little is known about its roles in mental health management and neuroplasticity. We conducted the first exploratory investigation evaluating the multimodal effects of a virtual sailing (VSail) program in healthy adults with the long-term goal of establishing its feasibility for mental health management.

**Methods:**

Here, we conducted a single-arm clinical trial in 24 healthy adults (18–64 years) who completed one 30-min VSail session weekly for 6 weeks using the VSail-Trainer^®^ simulator. Pre- and post-intervention assessments included motor function (hand grip strength, balance, and inertia) and mental health and clinical functioning self-report questionnaires (Beck Depression Inventory, Beck Anxiety Inventory, Perceived Stress Scale, Flanagan Quality of Life, Global Assessment of Functioning, and Health of the Nation Outcome Scales). Magnetic resonance spectroscopy analysis was performed in the ventromedial prefrontal cortex to evaluate the response of key neurometabolites [gamma-aminobutyric acid (GABA) and glutamate] to the VSail exercise program.

**Results:**

We found that the VSail program significantly enhanced grip strength in both dominant and non-dominant hands, and body balance. Hand inertial measurements across three axes showed no significant changes, indicating stable motor control and complexity. Mental health, clinical functioning, and neurometabolite levels remained stable, reflecting ceiling effects typical of healthy populations. Together, the results demonstrate that VSail effectively enhances motor performance while maintaining psychological and neurometabolic stability in healthy adults.

**Conclusions:**

Our findings indicate that virtual reality (VR)-based exercise, such as VSail, possesses strong potential as a safe, engaging, and scalable digital exercise strategy for future clinical applications, paving the way for understanding the role of virtual exercise in the muscle–brain axis.

## Introduction

Exercise plays a central role in maintaining and improving physical, cognitive, and overall wellbeing (Biddle et al., 2019). Current public health guidelines recommend that adults engage in at least 150 min of moderate-intensity or 75 min of vigorous-intensity physical activity per week to achieve meaningful health benefits (World Health Organization, 2023). Regular physical activity has been associated with a wide range of neurobiological adaptations, including structural and functional brain changes, improved cerebral blood flow, and enhanced neural network efficiency (Stillman et al., 2016). Although these mechanisms have been extensively studied, the specific neurochemical pathways through which exercise influences brain function in humans remain an active area of investigation.

Despite the well-established benefits of physical activity, participation in conventional exercise programs has limitations, particularly among individuals experiencing physical or psychological health challenges (Stubbs et al., 2018). Common barriers include fatigue, low motivation, pain, physical limitations, environmental constraints, and negative prior exercise experiences (Glowacki et al., 2017; Stubbs et al., 2018). These barriers can reduce adherence to traditional exercise interventions and contribute to disparities in physical activity participation. Consequently, alternative exercise approaches that are adaptable, engaging, and accessible across diverse populations are increasingly being explored (McKenna et al., 2024).

One promising avenue is virtual reality (VR)-based exercises. VR exercises have emerged as adaptable, immersive, and scalable platforms that combine the physiological benefits of physical activity with the motivational and cognitive engagement of interactive environments (Mocco et al., 2024). These digitally simulated movement-based activities allow users to perform physical tasks within immersive environments, providing real-time sensory feedback and adjustable task difficulty (Skjæret et al., 2016) and offering safe, cost-effective, and accessible alternatives to traditional exercise, particularly for populations with mobility limitations or mental health needs (Sarai et al., 2025). Indeed, VR training has been shown to promote balance, motor control, cognitive engagement, and emotional wellbeing while reducing common barriers to participation (Dockx et al., 2016).

Virtual sailing (VSail) has recently emerged as a rehabilitation program for people with physical disabilities, such as spinal cord injury (Manzanares et al., 2024), representing a novel VR-based exercise designed to simulate the motor and cognitive demands of real-world sailing. Increasing evidence suggests that sailing, as a recreational sport, offers therapeutic benefits for people with neurodegenerative diseases (Broadbent and Swalwell, 2020), severe spinal cord injury (Rojhani et al., 2017), severe mental disorders (Carta et al., 2014; Sancassiani et al., 2017), and post-traumatic stress disorder (PTSD) (Zabag et al., 2020). Sailing involves low-intensity exercise integrating motor-cognitive activity, requiring continuous adjustments in posture, balance, visuomotor coordination, decision-making, and situational awareness (Bera et al., 2021). However, implementing physical sailing clinically is limited by cost and safety concerns. In this context, VSail represents an exciting alternative, providing a controlled, accessible alternative, with early studies indicating improvements in balance, motor coordination, and psychosocial functioning (Xiao, 2025). Despite these promising findings, there is a lack of normative data on VSail’s effects in healthy adults across motor, psychological, and neuroplasticity domains. This presents a clear research gap in the development of virtual exercise-based interventions. Therefore, obtaining these baseline data is critical for informing future clinical implementation in the management of mental and physical health conditions.

To address this gap and, importantly, establish the feasibility of the VSail exercise program for mental health management, we conducted the first exploratory study aimed at investigating the effects of a 6-week VSail program in healthy adults. This study comprises multimodal outcome measures including motor performance (hand grip strength, balance, and inertial metrics), mental health and overall functioning, and key neurometabolites [gamma-aminobutyric acid (GABA) and glutamate], providing foundational normative data and laying the groundwork for future clinical studies that explore virtual exercise, such as VSail, as a new non-pharmacological exercise strategy. Specifically, this exploratory study aimed to determine whether a 6-week VSail program is sufficient to improve motor function and the overall wellbeing in a healthy population, and to assess whether ceiling effects are present in specific domains, such as mental health and neuroplasticity. Based on prior literature on VR-based motor training and exercise-induced neuroplasticity, we hypothesize that the VSail intervention would be associated with improvements in motor performance, while psychological and neurometabolic measures could remain stable in a healthy population due to expected ceiling effects. These exploratory findings aim to provide foundational data to inform future studies investigating the therapeutic potential of VSail in clinical populations.

## Methods

### Study design

To explore the feasibility and preliminary effects of a VSail exercise intervention in healthy adults, a single-arm pilot trial was conducted at Swinburne University of Technology, Victoria, Australia, from January 2024 to October 2025. The study was conducted in accordance with the Guidelines for Good Clinical Practice and was approved by the Swinburne University of Technology Human Research Ethics Committee (#20247579). Informed written consent has been obtained from all participants as per the approved research ethics.

### Participants

A total of 24 healthy adults aged 18 to 64 years (male or female) were recruited on a voluntary basis via advertising flyers, word-of-mouth, and social media. Participants were included if they did not have a self-report history or present neuropsychiatric or musculoskeletal diagnosis, cognitive impairment, uncorrected visual impairment, or any other condition that may impede performance. Participants were excluded if they did not meet the inclusion criteria, did not meet the fitness requirement assessed via the Adult Pre-Exercise Screening System, or had participated in another exercise therapy in the past 2 years (to avoid intervention contamination), or if they had insufficient English proficiency to successfully conduct assessments. All experimental protocols were thoroughly explained to each participant and informed consent was obtained prior to commencing the study. Participants who volunteered for the magnetic resonance spectroscopy (MRS) component underwent a safety screening conducted by a licensed radiographer and provided additional written consent following each magnetic resonance imaging (MRI) scan.

### VSail program

All participants were included in the VSail program, which consisted of six individual 30-min sessions, with one session per week for six consecutive weeks. A recent systematic and meta-analytic review demonstrated that 6 weeks was an adequate duration to show meaningful change in conventional and virtual-based exercises (Zhang et al., 2025). Each session was delivered via the VSail-Trainer^®^ simulator (Alvarabie et al., 2025) under the supervision of an exercise therapist, who provided verbal and tactile guidance to support participants and ensure their safety throughout the program. The sailing task was structured to replicate core elements of sailing, including continuous adjustment to environmental feedback through boat orientation, guided by a standardized computerized sailing program integrated in the VSail-Trainer^®^ simulator (Alvarabie et al., 2025). An individual session was composed of five 2- to 4-min trials, during which participants were required to navigate through one whole diamond-shaped route using the most efficient sailing strategy to counteract wind and course conditions. The wind and course conditions were maintained across all sessions and participants were provided 1-min rest period between each trial.

The intervention followed a fully standardized protocol across all participants, with identical session duration, task structure, and environmental conditions. No modifications to task difficulty or environmental parameters were introduced, ensuring that all participants were exposed to the same conditions throughout the intervention. The wind direction and environmental conditions were kept constant across all sessions, ensuring a controlled and standardized task environment. These demands required sustained attention, visual motor coordination, and real-time decision-making. Each session lasted approximately 30 min, with participants completing six sessions over the intervention period. All participants received the same instructions and an initial familiarization phase to ensure consistency in task understanding and execution. This standardized approach was implemented to enhance reproducibility and to ensure that any observed changes in motor or behavioral outcomes could be attributed to participant interaction with the intervention, rather than variability in task design.

### Outcome measures

The following validated outcome measures were all administered pre- and post-intervention via a standardized order as described below. Primary outcomes included motor function and coordination, mental health, and quality of life (QoL), while secondary outcomes included key neurometabolites such as GABA and glutamate. Given that the study population consisted of neurologically and psychiatrically healthy adults, these measures were not intended to detect clinical symptom changes but rather to ensure that the intervention did not adversely affect psychological wellbeing. Consequently, these variables were treated as exploratory outcomes rather than primary efficacy measures.

### Motor function

The motor function outcome measures comprised hand grip strength (dominant and non-dominant hands), body static balance performance, and hand inertia-based motion control across three axes. Maximal isometric handgrip strength was assessed via a JAMAR^®^ hydraulic hand dynamometer (0–90 kg dual scale), a validated and widely applied tool for evaluating upper extremity function and whole-body force production. Participants conduct three grip strength repetitions per hand before and after each session. The mean of these repetitions was recorded for the dominant and non-dominant hand. Static balance was measured using the Balance Error Scoring System (BESS), a quick clinical assessment involving three stance conditions (parallel, single-leg, and tandem) conducted on a firm and foam surface. For each stance, participants stood with hands on hips and eyes closed, maintaining each stance for 20 s, during which the number of errors was recorded. Deviations from the original stance, such as loss of balance, opening of eyes, or limb movement, were considered errors, with a maximum of 10 errors per stance. Only the firm surface condition was used for this study, with a total score ranging from 0 to 30 errors, where lower scores indicated better balance whereas higher scores indicated poor balance.

Motor performance and movement quality were quantified using Inertial Measurement Units (IMUs), which captured tri-axial acceleration data along the *X* (mediolateral), *Y* (anteroposterior), and *Z* (vertical) axes during pre- and post-intervention assessments. Data acquisition was conducted under standardized conditions to ensure reliability and repeatability of motion capture. Four principal parameters were derived from the IMU signals to characterize the magnitude, variability, and complexity of motor behavior via average acceleration magnitude (AAM), root mean square acceleration (RMSA), range, and approximate entropy of velocity magnitude (VmApEn) over each axis (*X*, *Y*, *Z*). AAM is calculated as the mean of the vector magnitude of tri-axial acceleration, with higher values reflecting more vigorous or dynamic motion, while lower values indicate reduced movement amplitude. AAM was computed for each axis (*X*, *Y*, *Z*) independently to assess directional contributions to movement control (Moe-Nilssen, 1998). RMSA quantifies the variability and smoothness of movement by capturing the magnitude of oscillations in acceleration (Kavanagh et al., 2004). Range values were extracted for each axis to assess directional motor adaptation (Lugade et al., 2014). VmApEn is calculated using the algorithm proposed by Pincus (1991), and ranges between 0 and 2. Entropy was computed for each axis (*X*, *Y*, *Z*) to evaluate changes in neuromotor complexity following the VSail program (Stergiou and Decker, 2011).

### Mental health, quality of life, and clinical functioning

This study was conducted as an exploratory study to establish the feasibility of the VSail exercise program for future mental health management, accordingly, participants’ overall wellbeing, mental health, and QoL were assessed with a series of validated self-report questionnaires, including the Beck Anxiety Inventory (BAI), Beck Depression Inventory (BDI), Perceived Stress Scale (PSS), Flanagan Quality of Life Scale (QoLS), Global Assessment of Functioning (GAF) Scale, and Health of the Nation Outcome Scales (HoNOS) at baseline (pre-intervention) and after the last sailing session (post-intervention). The Flanagan QoLS is a validated multidimensional tool consisting of 16 items that cover five broad life domains, including the following: material and physical wellbeing, relationships, personal development and fulfilment, and social, community, and civic activities (Burckhardt and Anderson, 2003). The BAI and BDI are each 21-item self-reported instruments designed to evaluate the severity of anxiety and depression symptoms, respectively, over the past 2 weeks (Lee et al., 2018). Participants rate each item on a four-point Likert scale, and these ratings are summed so that higher scores indicate more severe symptoms, while lower scores indicate milder symptoms. The abridged PSS is a 10-item self-reported scale that measures perceived stress over the past 2 weeks via a five-point Likert scale, with higher summed scores indicating greater perceived stress and lower scores indicating less perceived stress (Harris et al., 2023).

### Neurometabolites

To evaluate the impact of the VSail program on neuroplasticity, concentrations of glutamate (the major excitatory neurotransmitter) and GABA (the major inhibitory neurotransmitter) were measured using MRS conducted on a Siemens Trio 3T MRI Scanner at the Swinburne University of Technology. As an exploratory study to establish the VSail feasibility for future mental health management, the ventromedial prefrontal cortex (vmPFC), a region critical to mood and cognitive regulation (Alexander et al., 2023), was selected for MRS analysis. Participants in this study underwent an MRI scan adopting a published protocol (Alvarabie et al., 2025). A T1-weighted magnetization-prepared rapid acquisition gradient echo (MPRAGE) anatomical image was acquired for voxel placement and tissue segmentation (TR = 2,300 ms, TE = 2.07 ms, voxel size = 1 mm^3^, duration = 3.52 min). A Hadamard encoded MEGA-edited PRESS sequence (HERMES) was subsequently used to enhance metabolite signals in vmPFC (TR = 2,000 ms, TE = 80 ms, editing pulses at 1.9 and 7.5 ppm, 50 Hz bandwidth, ON–OFF averages, scan duration = 10.48 min). Eight ON–OFF averages of unsuppressed water were acquired via identical parameters. A 20 × 25 × 20 mm^3^ voxel was co-registered to the T1-weighted image and segmented via Gannet, which used Statistical Parametric Mapping (SPM12, Wellcome Trust Centre for Neuroimaging, London, UK) to estimate voxel fraction in gray matter, white matter, and cerebrospinal fluid. MRS data were pre-processed and quantified via MATLAB R2024b (The Mathworks, Natick, MA, USA) and associated toolboxes. The direction of neurometabolite modulation was interpreted relative to baseline values, representing return toward homeostatic equilibrium.

### Statistical analysis

A power analysis was conducted using G-Power version 3.1.9.7, finding that sample sizes of 24 and 16 are sufficient to detect moderate to large changes (*d* = 0.52 and *d* = 0.65, respectively) from pre- to post-intervention with 80% power when using a one-sided test and a significance level of 5%. Data were analyzed using IBM SPSS Statistics for Windows, Version 30.0.0.0 (IBM Corp., Armonk, NY, USA). Normal distribution of data was assessed via the Shapiro–Wilk test and Q–Q plots. All results are expressed as mean and standard deviation unless otherwise specified. Statistical significance was set at *p* < 0.05 for all tests. A paired-sample *t*-test was conducted to determine if a 6-week VSail program significantly improved motor function, mental health, QoL, and neurometabolite outcomes among a healthy sample of 24 or 16 adults (**Table 1**) with a Bonferroni correction applied in order to avoid false positives from multiple tests. All IMU parameters (AAM, RMSA, Range, and VmApEn) were computed separately for the *X*, *Y*, and *Z* axes, and again compared pre- and post-intervention with a Bonferroni correction for multiple tests. This multidimensional IMU-based analysis enabled a comprehensive evaluation of movement magnitude, stability, amplitude, and neuromotor adaptability in response to the VSail program. In view of the small sample sizes and the use of paired *t*-tests, no attempt was made to control for age or sex in the analysis.

**Table 1:** Participant demographics.

Variables	Men (*n* = 14)	Women (*n* = 10)	Total (*n* = 24)
Age (years)	29.571 (8.751)	32.600 (7.214)	30.833 (8.122)
BMI (kg/cm^2^)	24.490 (3.780)	25.900 (5.662)	25.071 (4.535)
Handedness (*n*, %)			
Right	12 (86)	10 (100)	22 (92)
Left	2 (14)	0 (0)	2 (8)
Assessment completion (*n*, %)			
Motor assessments (*n* = 24)	14 (100)	10 (100)	24 (100)
MDD-QoL questionnaires (*n* = 16)	9 (64)	7 (70)	16 (67)
Neurometabolite (MRS) (*n* = 16)	9 (64)	7 (70)	16 (67)

Data are expressed as mean and standard deviation, unless otherwise indicated by sample number and percentage. BMI, body mass index; MDD, major depressive disorder; MRS, magnetic resonance spectroscopy.

## Results

### Characteristics of participants

Twenty-four healthy adults (14 men and 10 women) with a mean age of 30.83 ± 8.12 years participated in this study (**Table 1**). There were no dropouts, resulting in a 100% adherence rate across all sessions and motor assessments. A total of 16 participants (67%) completed the QoLS, BAI, BDI, PSS, and MRS assessments at baseline and post-intervention timelines. No incidents of cybersickness, motion sickness, falls, injuries, or other adverse events or discomfort were reported during the study period from all 24 participants.

### Motor function

**Figure 1:**
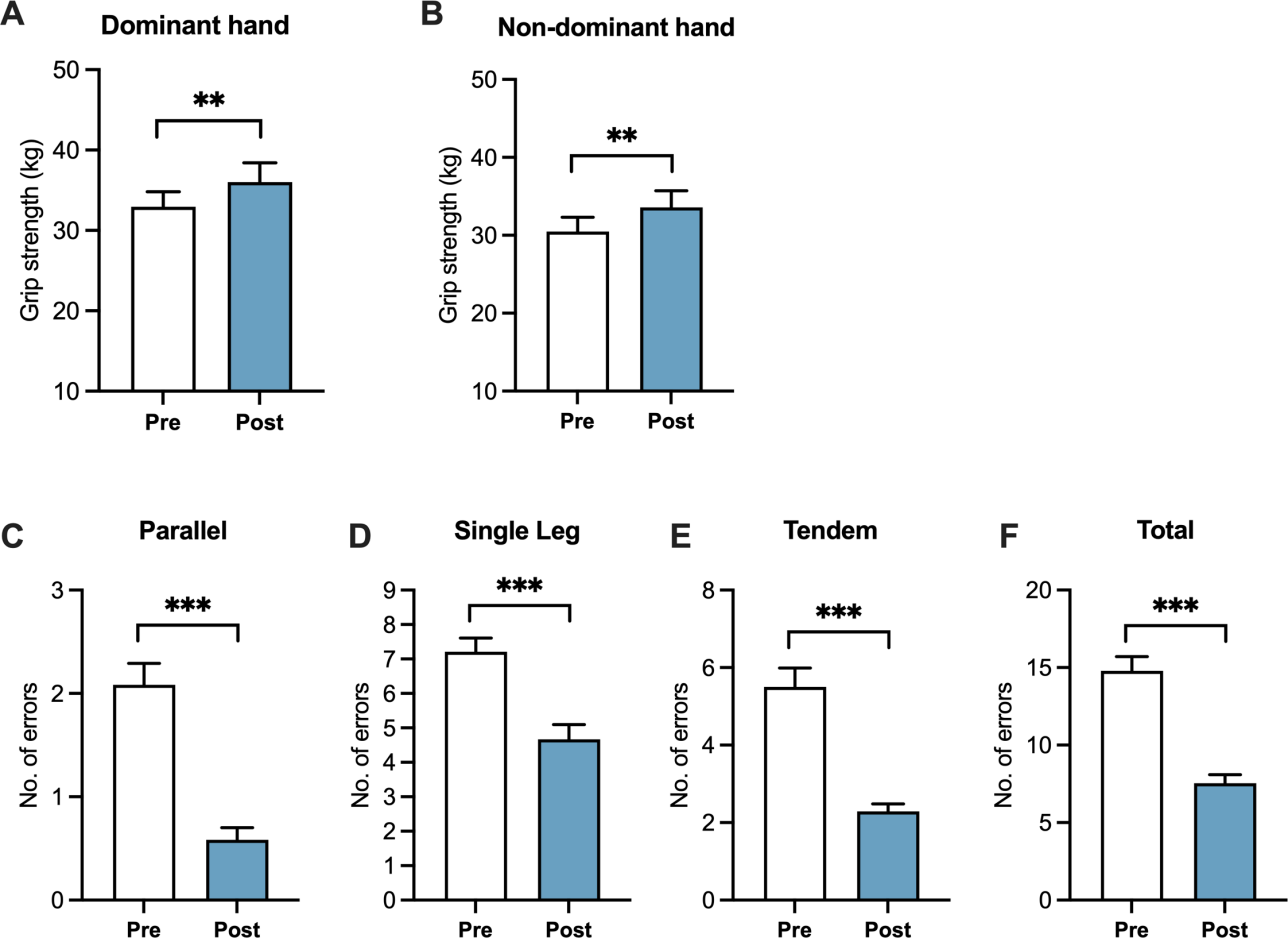
Analysis of hand grip strength and body balance. **(A, B)** Analysis of grip strength over time for dominant and non-dominant hands. Changes in grip strength (kg) from pre- to post-intervention for the dominant **(A)** and non-dominant **(B)** hands, as measured using the JAMAR dynamometer. Data = mean ± standard error of the mean. **(C–F)** Analysis of the number of errors from pre- to post-intervention for the parallel **(C)**, single-leg **(D)**, tandem **(E)**, and total **(F)** stances, as measured by the Balance Error Scoring System (BESS). Data = mean ± standard error of the mean. A paired *t-*test was conducted on a sample of *n* = 24. ***p < 0.001.

**Figure 2:**
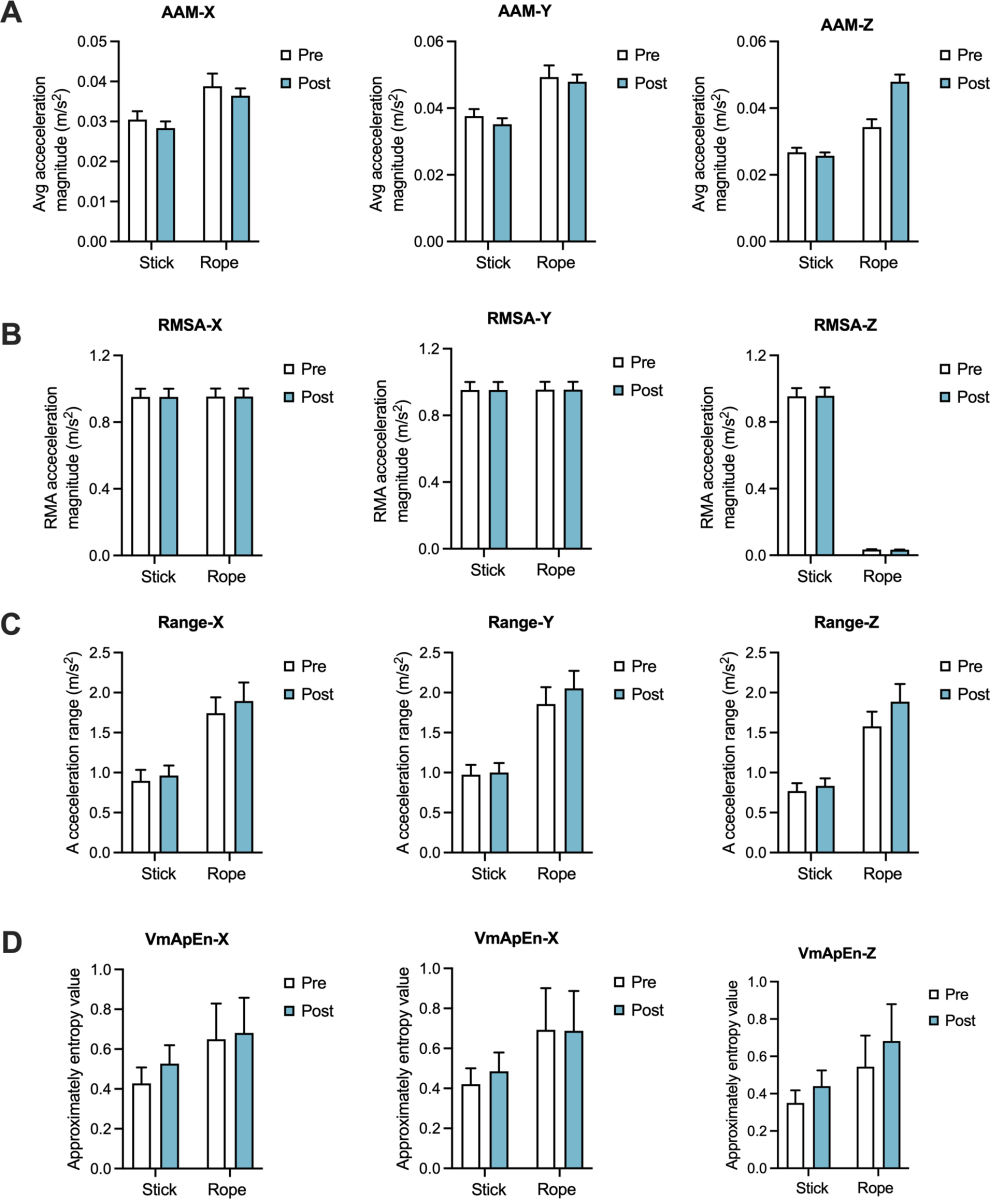
Analysis of inertia. Changes in four inertia scores (AAM, RMSA, Rg, and VmApEn) for the right hands that hold the stick and the left hand that holds the rope at pre- and post-intervention. **(A–C)** AAM of three different axes: *X*, *Y*, and *Z*. **(D–F)** RMSA of the three different axes *X*, *Y*, and *Z*. **(G–I)** Range of the three different axes: *X*, *Y*, and *Z*. **(J–L)** VmApEn of the three different axes: *X*, *Y*, and *Z*. Data, mean ± standard deviations. A one-way ANOVA was conducted on a sample of *n*, 24. AAM, average acceleration magnitude; RMSA, root mean square acceleration; Rg, range; VmApEn, approximate entropy of velocity magnitude (a paired *t-*test, *n* = 24).

Participants (*n* = 24) demonstrated significant improvements in hand grip strength and balance performance following the VSail intervention (**Table 2**, **Figures 1**, **2**). Dominant-hand grip strength significantly increased from pre- to post-intervention [*M*_pre_ = 32.963 ± 9.111 kg; *M*_post_ = 36.022 ± 11.756 kg; *t*(23) = −3.553, *p* = 0.002, *d* = −0.725] (**Figure 1A**), with a similar increase also demonstrated for the non-dominant hand [*M*_pre_ = 30.482 ± 8.978 kg; *M*_post_ = 33.571 ± 10.536; *t*(23) = −4.247, *p* < 0.001, *d* = −0.701] (**Figure 1B**). Significant reductions in the number of balance errors were also observed across all BESS conditions. Total BESS errors decreased from pre- to post-intervention [*M*_pre_ = 14.792 ± 4.482 errors; *M*_post_ = 7.542 ± 2.734 errors; *t*(23) = 8.407, *p* < 0.001, *d* = 1.716] (**Figure 1D**). Improvements were also demonstrated within individual stance conditions, including parallel stance [*M*_pre_ = 2.083 ± 1.018 errors; *M*_post_ = 0.583 ± 0.584 errors; *t*(23) = 7.514, *p* < 0.001, *d* = 1.534] (**Figure 1C**), single-leg stance [*M*_pre_ = 7.208 ± 1.978 errors; *M*_post_ = 4.667 ± 2.099 errors; *t*(23) = 5.198, *p* < 0.001, *d* = 1.061] (**Figure 1D**), and tandem stance [*M*_pre_ = 5.500 ± 2.414 errors; *M*_post_ = 2.292 ± 0.955 errors; *t*(23) = 7.188, *p* < 0.001, *d* = 1.467] (**Figure 1E**). No significant change was observed for any inertia metrics across all axes (**Figure 2**).

**Table 2:** Outcome measurements.

Variables	Pre-intervention	Post-intervention	Mean change	*p*-value	Cohen’s *d* effect size
BESS (number of errors) (*n* = 24)					
Parallel stance	2.083 (1.018)	0.583 (0.584)	−1.500***	<.001	1.534
Single-leg stance	7.208 (1.978)	4.667 (2.099)	−2.542***	<.001	1.061
Tandem stance	5.500 (2.414)	2.292 (0.955)	−3.208***	<.001	1.467
Total	14.792 (4.482)	7.542 (2.734)	−7.250***	<.001	1.716
Grip strength (kg) (*n* = 24)					
Dominant hand	32.963 (9.111)	36.022 (11.756)	3.060***	0.002	−0.725
Non-dominant hand	30.482 (8.978)	33.571 (10.536)	3.090***	<.001	−0.701
Inertia metrics (*n* = 24)					
AAM (m/s^2^)					
AAM *X* stick	0.030 (0.0091)	0.028 (0.0075)	−0.002	0.7 30	0.373
AAM *X* rope	0.039 (0.0141)	0.036 (0.0081)	−0.002	0.816	0.200
AAM *Y* stick	0.038 (0.0095)	0.035 (0.0081)	−0.003	0.69	0.159
AAM *Y* rope	0.049 (0.0157)	0.048 (0.0095)	−0.001	0.947	0.068
AAM *Z* stick	0.027 (0.0060)	0.026 (0.0044)	−0.001	0.827	0.081
AAM *Z* rope	0.034 (0.0104)	0.034 (0.0066)	0	0.995	0.019
RMSA (m/s^2^)					
RMSA *X* stick	0.952 (0.2181)	0.952 (0.2190)	0	0.999	−0.066
RMSA *X* rope	0.953 (0.2151)	0.954 (0.2130)	0.001	0.999	−0.293
RMSA *Y* stick	0.953 (0.2157)	0.952 (0.2151)	0	0.999	0.099
RMSA *Y* rope	0.954 (0.2110)	0.954 (0.2123)	0	0.999	0.212
RMSA *Z* stick	0.955 (0.2198)	0.958 (0.2206)	0.003	0.999	−0.413
RMSA *Z* rope	0.034 (0.0104)	0.033 (0.0068)	−0.001	0.952	0.073
Range (m/s^2^)					
Rg *X* stick	0.897 (0.613)	0.963 (0.558)	0.065	0.941	−0.312
Rg *X* rope	1.743 (0.886)	1.896 (1.034)	0.152	0.885	−0.422
Rg *Y* stick	0.975 (0.543)	1.002 (0.527)	0.027	0.988	−0.171
Rg Y rope	1.858 (0.941)	2.053 (0.978)	0.196	0.818	−0.482
Rg *Z* stick	0.955 (0.437)	0.958 (0.416)	0.003	0.895	−0.280
Rg *Z* rope	1.579 (0.820)	1.886 (0.990)	0.307	0.579	−0.702
VmApEn (units)					
VmApEn *X* stick	0.428 (0.357)	0.527 (0.413)	0.099	0.725	−0.377
VmApEn *X* rope	1.743 (0.802)	1.896 (0.791)	0.152	0.992	−0.128
VmApEn *Y* stick	0.421 (0.357)	0.485 (0.422)	0.064	0.877	−0.305
VmApEn *Y* rope	0.693 (0.934)	0.688 (0.892)	−0.005	0.999	0.080
VmApEn *Z* stick	0.350 (0.304)	0.440 (0.378)	0.090	0.716	−0.353
VmApEn *Z* rope	0.545 (0.743)	0.682 (0.884)	0.137	0.872	−0.384
Mental health metrics (*n* = 16)					
QoLS	93.063 (14.406)	94.938 (14.341)	1.875	0.544	−0.155
PSS	25.125 (12.727)	25.188 (12.210)	0.063	0.987	−0.004
BDI	3.875 (4.145)	3.562 (4.676)	−0.313	0.771	0.074
BAI	4.438 (5.329)	5.500 (6.303)	1.063	0.298	−0.270
Overall functioning metrics (*n* = 16)					
HoNOS	1.750 (2.082)	2.938 (5.397)	0.563	0.368	−0.232
HoNOS—other mental and behavioral problem	1.000 (1.648)	1.867 (3.758)	0.813	0.370	−0.239
GAF	93.563 (6.870)	94.375 (5.071)	0.813	0.634	−0.122
Neurometabolite concentration (CSF-corrected iu) (*n* = 16)					
GABA	4.655 (7.677)	7.188 (12.602)	2.533	0.529	−0.161
Glx	8.850 (3.193)	8.803 (1.210)	−0.047	0.953	0.015
% of GABA	30.5	48.8	0.183	0.596	−0.174
% of Glx	58.0	59.5	0.015	0.782	−0.070

Data expressed as mean and standard deviation unless otherwise specified. BESS, Balance Error Scoring System; AAM, average acceleration magnitude; RMSA, root mean square; Rg, range; VmApEn, approximate entropy of velocity magnitude; QoLS, Flanagan Quality of Life Scale; PSS, Perceived Stress Scale; BDI, Beck Depression Inventory; BAI, Beck Anxiety Inventory; GAF, Global Assessment of Functioning; HoNOS, Health of the Nation Outcome Scales; GABA, gamma-aminobutyric acid; Glx, glutamate–glutamine; CSF, cerebrospinal fluid; iu, institutional units (paired-sample *t*-test followed by a Bonferroni correction, ***p < 0.001).

### Mental health, quality of life, and clinical functioning

**Figure 3:**
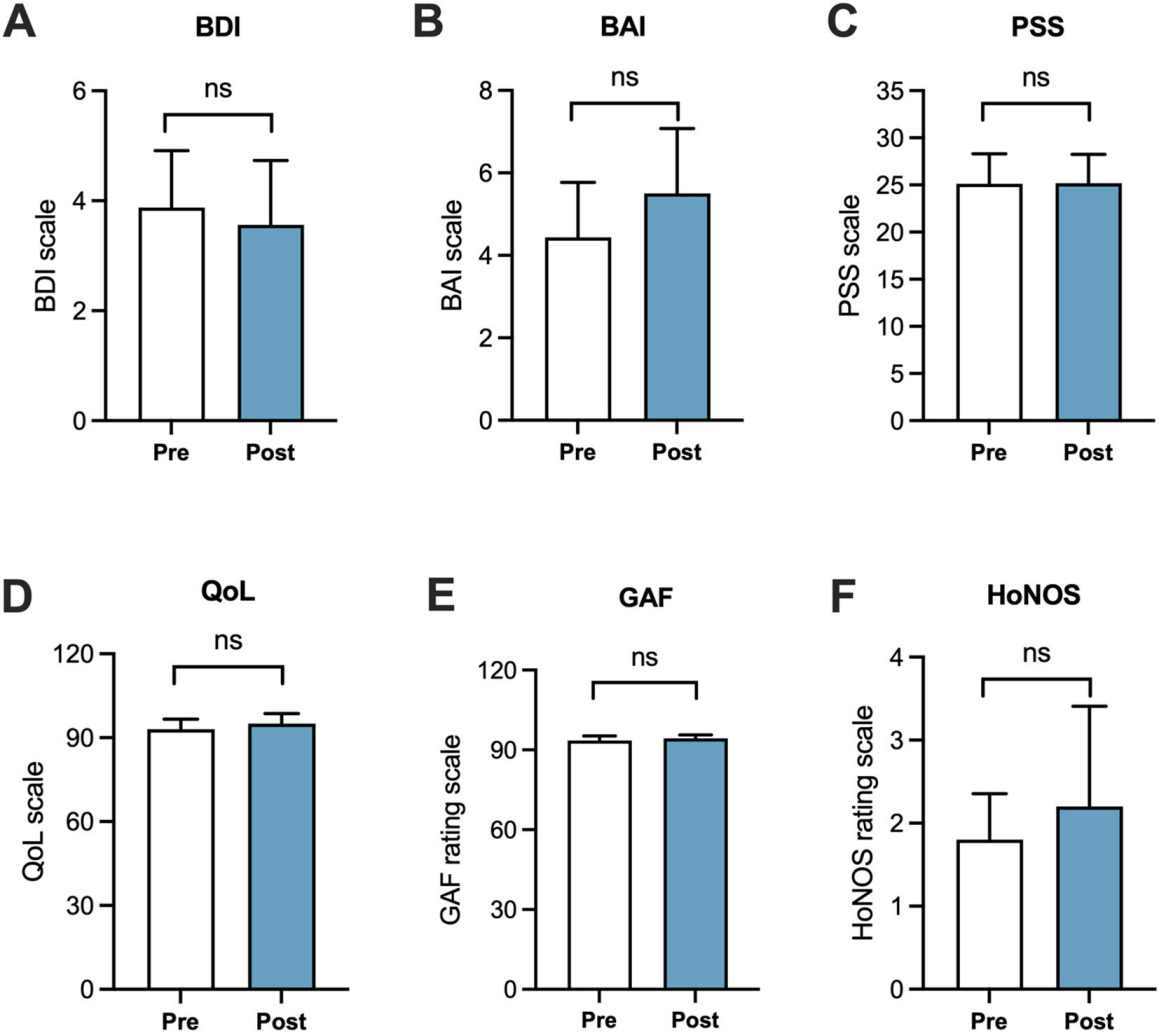
Analysis of mental health and quality of life. Changes in quality of life relating to mental health, including Beck Depression Inventory [BDI, **(A)**], Beck Anxiety Inventory [BAI, **(B)**], Perceived Stress Scale [PSS, **(C)**] and Quality of Life [QoL, **(D)**], Global Assessment of Functioning [GAF, **(E)**], and Health of the Nation Outcome Scales [HoNOS, **(F)**] from pre- to post-intervention. Data = mean ± SEM. A paired *t-*test was conducted on a sample of *n* = 16.

**Figure 4:**
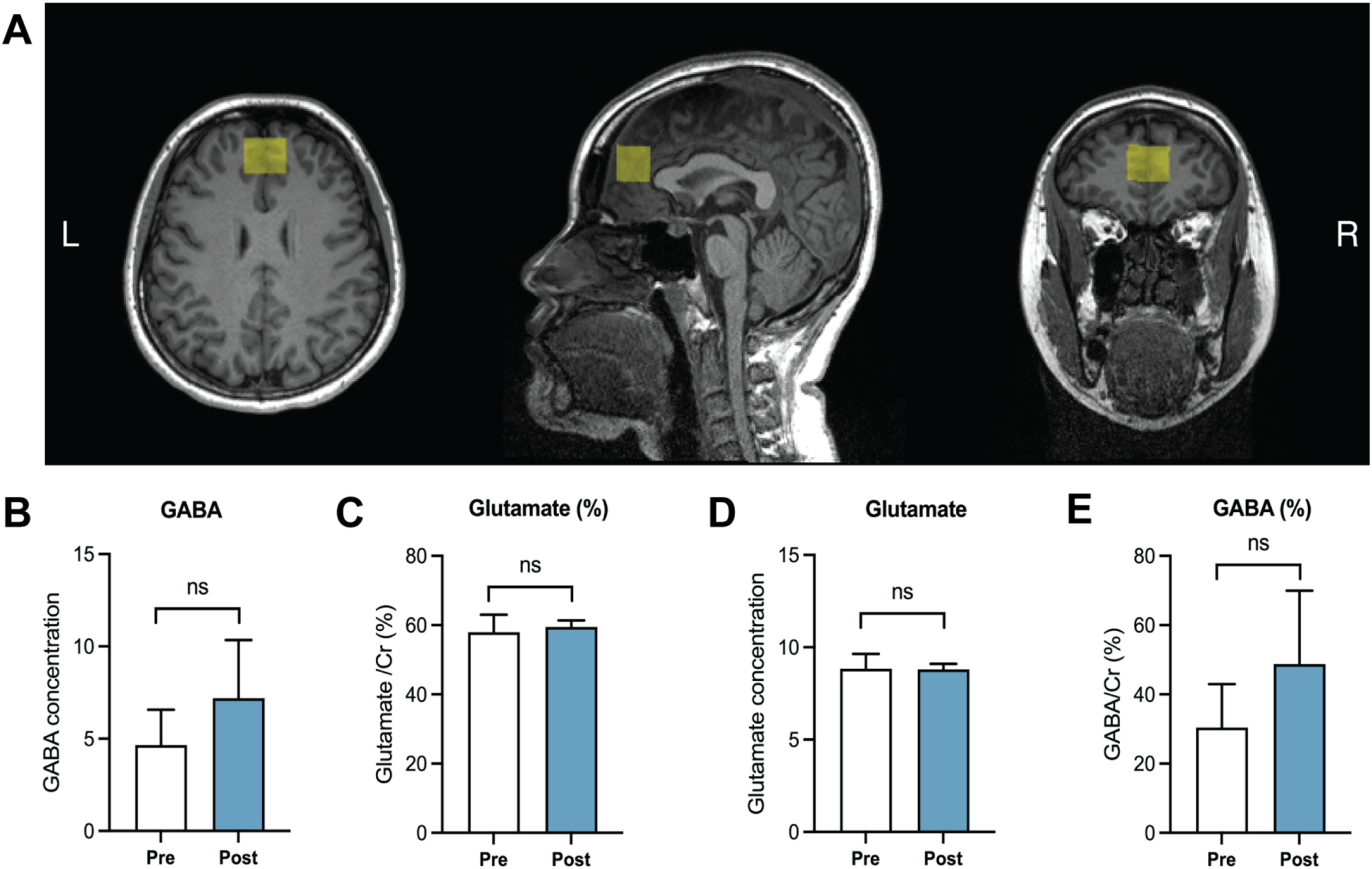
Magnetic resonance spectroscopic analysis of key neurometabolites. **(A)** Representative images over three planes showing the voxel (labelled with a yellow box) of the ventromedial prefrontal cortex (vmPFC) used for analyzing key neurometabolites via magnetic resonance spectroscopy imaging. **(B, C)** Analysis of gamma-aminobutyric acid (GABA) concentration **(B)** and ratio [relative to creatine, **(C-E)**] Analysis of glutamate concentration **(D)** and ratio [relative to creatine, **(E)**]. Data = mean ± standard deviations. A paired *t-*test was conducted on a sample of *n* = 16.

Mental health and QoL were measured using a series of validated self-report questionnaires. The overall mental health and QoL status remain steady in participants (*n* = 16) who completed the full assessments, and no statistically significant changes were observed from pre- to post-intervention (**Table 2**, **Figures 3**, **4**). The consistent scores across several domains assessed via a combination of subjective (BDI, BAI, PSS, QoLS, and HoNOS) and clinician-rated (GAF) questionnaires indicate that the VSail intervention was well tolerated, with no adverse impact on mental health or functioning in the healthy cohort, supporting the feasibility and safety of VSail as a potential therapeutic exercise platform for future clinical populations.

### Neurometabolites

Having evaluated mental health status via self-report questionnaires, MRS was used to analyze concentrations of two major neurometabolites (GABA and glutamate) in the vmPFC (**Figure 4A**), a region involved in mood change and executive function. Similar to the mental health data (**Figure 3**), no significant changes were observed in the concentrations or creatine-normalized percentages of GABA and glutamate in the vmPFC region following the intervention in 16 participants who completed the full MRI (**Table 1**, **Figure 4**). Overall, the MRS data demonstrate that the 6-week VSail program exerted no significant impact on GABA or glutamate concentrations within the cortical region associated with mood change, suggesting that repeated VSail exposure exerts minimal effect upon the neurometabolite profiles in the healthy population.

## Discussion

This is an exploratory study that, for the first time, evaluated a 6-week VSail exercise program among a cohort of 24 healthy adults, integrating multimodal assessments of motor performance, mental health, QoL, and neurometabolic status, establishing the trial’s feasibility. The findings demonstrate that VSail elicits significant improvements in some but not all motor function domains, such as grip strength and static balance, while maintaining stable mental health and neurochemical profiles. This is the first study that integrated motor assessments, mental health evaluation, and neuroimaging within a virtual exercise intervention, establishing the scientific basis essential for future clinical translation.

The only significant outcomes following VSail intervention in healthy adults occurred in the motor domain, with significant gains in bilateral hand grip strength and reductions in BESS error scores, indicating better postural stability and sensorimotor integration. These results align with evidence that immersive, feedback-based motor activities enhance neuromuscular coordination, proprioception, and balance (Hajder et al., 2025; Lee et al., 2024). Grip strength and static balance are clinically relevant biomarkers that decline in conditions such as major depression, multiple sclerosis, frailty, and cardiometabolic disease, where deficits contribute to disability, slower cognition, and reduced QoL (Knapen et al., 2015). Bilateral strength gains also suggest interhemispheric transfer of motor learning, a mechanism reported in visuomotor training (Levac et al., 2019). Collectively, these findings indicate that VSail may offer a safe, engaging, and accessible adjunct exercise modality for addressing motor deficits in clinical populations facing barriers to traditional physical activity. However, given the single-arm design, these findings should not be interpreted as causal effects.

The dynamic visuomotor demands of VSail, requiring continuous adjustment of steering and rope handling, likely drove these improvements, consistent with cognitive–motor coupling theories emphasizing attention, feedback, and adaptability (Linares-Vargas and Cieza-Mostacero, 2024). In contrast, inertial parameters (AAM, RMSA, Rg, and VmApEn) showed no significant changes across *X*, *Y*, and *Z* axes, suggesting stable acceleration patterns. This may reflect efficient motor control with improved precision rather than power-based adaptation, aligning with the concept of “optimal variability,” where small, non-systematic fluctuations indicate healthy sensorimotor function (Stergiou and Decker, 2011). The dissociation between improvements in strength and balance and the absence of detectable changes in IMU-derived kinematic metrics provides important insight into the nature of motor control adaptations induced by the VSail intervention. While gains in balance performance suggest enhanced functional stability and postural control, the stability of IMU metrics indicates that these improvements were not achieved through rigidification or simplification of movement patterns. Instead, participants appear to have maintained their existing movement variability while becoming more effective at controlling it. Overall, these findings highlight the high adaptability of the VSail program.

An expected finding of this study is that mental health and overall functioning remained stable throughout the VSail program. Given the participants’ low baseline symptoms, substantial improvements are unlikely. Clinical scores (GAF and HoNOS) stayed within normal limits, confirming no adverse psychological impact. These findings support the safety and tolerability of VSail, consistent with similar VR-based wellness programs (Laver et al., 2017; Lee et al., 2024). Previous exergaming literature has shown that low-intensity, engaging virtual exercise maintains emotional stability and intrinsic motivation (Wang and Wang, 2025). Together with the literature, the results of this study suggest that while mood and stress did not change significantly, VSail may have helped maintain psychological wellbeing and life satisfaction. Importantly, the absence of deterioration underscores its safety and suggests potential for adaptation in clinical populations such as major depression, anxiety, or PTSD.

Consistent with the aforementioned mental health outcomes, neurometabolite levels remained relatively unchanged following the VSail program. GABA and glutamate in the vmPFC were stable, which is clinically relevant as disruptions in these systems are biomarkers of symptom severity and treatment response in disorders such as major depressive disorder (MDD) and anxiety (Lener et al., 2017). MRS-derived measures are increasingly used to monitor disease progression and intervention effects, including pharmacological and neuromodulatory therapies such as antidepressants, rTMS, ketamine, and structured exercise programs (Sarawagi et al., 2021). The absence of neurometabolic perturbation in healthy adults suggests that VSail does not induce excitatory overstimulation or inhibitory imbalance, a desirable characteristic for future implementation in clinical populations with already dysregulated neurochemical systems. Indeed, interventions that impose excessive cortical excitatory load may exacerbate symptoms in MDD or anxiety, whereas stable or gradually adaptive shifts are considered safer and more therapeutically promising. Given this is an exploratory study in healthy adults, the absence of significant changes in psychological and neurometabolic measures not only reflects the absence of underlying dysregulation in this cohort but also provides evidence of intervention safety, suggesting that participation in the VSail program did not adversely affect psychological wellbeing or neurochemical balance. However, these findings should be interpreted cautiously, as the study was not powered to detect subtle neurochemical changes.

Recent studies have shown that physical or cognitively engaging exercise can modulate GABAergic and glutamatergic pathways (Stagg et al., 2011), though such effects often require higher training intensity, longer duration, or larger sample sizes to detect meaningful neurometabolic change. Importantly, because altered GABA/glutamate ratios in the vmPFC have been linked to anhedonia, rumination, and treatment non-response in MDD (Duman et al., 2019), the stable profile observed here suggests that VSail may serve as a neurochemically safe adjunctive exercise modality capable of improving motor function and engagement without disrupting sensitive cortical inhibitory–excitatory dynamics. This safety profile, combined with the observed motor improvements, supports the potential of VSail as a promising rehabilitative tool for populations where neurometabolic imbalance is a core feature of the disorder and where behavioral activation is often limited.

A key contribution of this study is demonstrating multidomain responses to VSail in healthy adults. To our knowledge, this is the first VR exercise study that assessed neuroplasticity alongside motor and psychological outcomes. No prior work has simultaneously characterized motor performance, mental health, wellbeing, and neurometabolite profiles within a single virtual exercise intervention. By integrating these domains, our multimodal dataset provides comprehensive reference values that can be used to contextualize future findings when VSail is adopted as a therapeutic modality for clinical populations, particularly for conditions such as MDD, where motor, affective, and neurochemical impairments are common (Sanacora et al., 2003).

The findings of this study highlight the potential of virtual exercise to improve motor performance while maintaining psychological and neurochemical stability, a critical balance for clinical populations sensitive to overstimulation or stress. Building on this, VSail may serve as a primary or adjunct non-pharmacological intervention for conditions such as MDD or anxiety, where impairments in psychomotor functioning, motivational deficits, and neurometabolite dysregulation are common. The gamified and enjoyable nature of the VSail environment may be particularly advantageous for these groups, as it offers an engaging, low-impact platform that can increase motivation to participate in physical activity, an area where traditional exercise programs often struggle to achieve adherence. Practically, VSail offers a scalable, accessible solution that reduces barriers related to mobility, stigma, and confidence. By combining structured physical activity with immersive, reward-based experiences, VSail may promote sustained engagement and amplify therapeutic gains. Taken together, the findings support VSail as a safe, feasible, and promising virtual exercise intervention warranting further evaluation in controlled clinical trials.

### Limitations and future directions

Limitations exist in the study. This is a single-arm pilot study without a control group, hence limiting our conclusion relating to an intervention-specific effect. The small sample size, absence of a control group, and single-arm design limit the power analysis. Although the power analysis showed that there was sufficient power to detect moderate to large effect sizes, even after the application of Bonferroni corrections, the small sample size meant that only paired-sample *t*-tests could be justified, without consideration of any sex or age effects.

Participants were healthy adults with low baseline distress, introducing ceiling effects that may have masked psychological changes. In addition, 67% completed MRS and QoL assessments (pre- and post-intervention). Future research should employ randomized controlled trials with a larger sample size and expanded neuroimaging to explore structural and functional changes over time. Incorporating physiological biomarkers (e.g., cortisol and heart rate variability) may clarify autonomic–neurochemical links. Moreover, the relatively short intervention duration and the use of a healthy adult may have limited the sensitivity for detecting measurable neurochemical changes in the brain. Future studies investigating virtual exercise interventions focusing on motor outcomes may benefit from including MRS voxels within motor-related cortical regions to better capture potential neuroplastic adaptations associated with motor learning and sensorimotor integration. Importantly, testing VSail among individuals with a medical diagnosis such as major depression will determine its clinical efficacy in modulating both motor and emotional regulation networks.

## Conclusion

The current study demonstrates that VSail exercise significantly enhances motor performance while maintaining stable mental and neurochemical profiles in healthy adults. The observed gains in grip strength and balance, coupled with stable psychological and neurobiological indices, confirm the feasibility, safety, and potential therapeutic relevance of VSail for future clinical applications. Despite being an exploratory pilot study, this study provides new and original findings to the field through identifying that participation in an acute VSail program improves some domains of motor performance, while displaying potential ceiling effects in psychological and neurochemical outcomes of the healthy population. Therefore, the findings of this study, for the first time, provide original normative data on the multidimensional responses to VSail and support its feasibility as a virtual exercise modality. These findings establish a scientific framework for future investigations into its efficacy as a non-pharmacological digital exercise approach for people with neurological or neuropsychological conditions.

## Data Availability

The raw data supporting the conclusions of this article will be made available upon reasonable request to the corresponding author, without undue reservation.
